# Measuring Women's Decision‐Making Power Beyond Self‐Reports: Evidence on the Nutritional Status of Children in Benin

**DOI:** 10.1111/mcn.70277

**Published:** 2026-10-29

**Authors:** Emeline Jacqueline Tossou

**Affiliations:** ^1^ Center for Economic Research University of Abomey‐Calavi (CRE‐UAC) Abomey‐Calavi Atlantic Department Benin

**Keywords:** Benin, bivariate probit model, nutritional status of children, women's decision‐making power

## Abstract

In Benin, malnutrition is the main risk factor for mortality and morbidity among young children. Women's decision‐making power within the household appears to be a factor that promotes adequate nutrition for children, both in terms of quantity and quality. The objective of this study is to analyze the effect of women's decision‐making power within the household on the nutritional status of children under 5 in Benin. The data used come from the 2017‐2018 Benin Demographic and Health Survey, and a total of 4,093 children under the age of 5 were included in the analysis. We use the composite index of anthropometric failure as a measure of children's nutritional status because of its ability to estimate the full extent of child malnutrition in the population. Women's decision‐making power is measured using women's own responses and cross‐referenced responses from both spouses on household decision‐making. For the empirical modeling of the study, we use the recursive bivariate probit model to account for the endogeneity of women's decision‐making power. Overall, women's decision‐making power does not significantly reduce the probability of experiencing anthropometric failure among children, but it increases this probability when the husband grants decision‐making power to his wife. This type of power reflecting a disagreement between spouses regarding decision‐making within the household, undercuts, rather than improve women's ability to influence their children's nutritional status. The authority granted by the husband may involve a transfer of responsibility in a context of economic vulnerability. Thus, a woman may be recognized as a decision‐maker but still lack sufficient resources, namely the financial or material means, to ensure adequate food for her children or improve their nutritional status. Policies aimed at strengthening women's decision‐making power should be accompanied by mechanisms to improve access to and control over economic resources, so that this power may lead to better child nutritional status.

## Key Messages

- Cross‐referencing spouses' responses offers a more nuanced understanding of the link between women's decision‐making power and under 5 children's nutritional status.
- Women's decision‐making power regarding major household purchases and their own healthcare does not significantly reduce the probability of anthropometric failure (measured by the CIAF).
- The exception is when a husband grants his wife decision‐making power over major household purchases, which significantly increases this probability. This may be explained by responsibility being transferred without real control over the resources.
- Policies strengthening women's decision‐making power should be accompanied by mechanisms to improve access to, and control over, economic resources.

## 1 Introduction

Improving the health and nutritional status of children under the age of five is a major development objective that interacts with other dimensions, such as education. Indeed, good health and adequate nutrition in preschool age promote the assimilation of academic knowledge during childhood and adolescence and improve productivity in adulthood (Alderman et al. 2006; UNICEF 2021b). Conversely, nutritional problems such as stunting, underweight and wasting have negative short‐, medium‐, and long‐term effects on human development (Assaf and Pullum 2018; Walker et al. 2007). Nutrition in utero, during the first 24 months of life, and throughout early childhood is essential for healthy growth (Victora et al. 2010).

In 2020, an estimated 149.2 million (22%) of the world's children under 5 were stunted, 45.4 million (6.7%) were wasted, and 38.9 million (5.7%) were overweight (UNICEF, WHO, The World Bank Group 2021). In addition, Africa alone accounts for 41% of stunted children, 27% of wasted children and 27% of overweight children (UNICEF, WHO, The World Bank Group 2021). In Benin, malnutrition is the main risk factor for mortality and morbidity among young children (UNICEF 2021a). Data from the fifth Demographic and Health Survey of 2018 reveal that only 15% of children aged between 6 and 23 months receive a diet that complies with recommendations on breastfeeding, dietary diversity and meal frequency (INSAE & ICF International 2019). In addition, around a third of children under the age of 5 are stunted or chronically malnourished, and around 7 out of 10 children aged 6 to 59 months suffer from anemia (INSAE & ICF International 2019).

Several factors identified in the literature play an important role in the nutritional outcomes of children in low‐income countries. Some studies highlight maternal characteristics as key determinants (Black et al. 2013; Duflo 2012a; Shroff et al. 2009). For example, low maternal body mass index (BMI) and abnormal height increase the risk of low birthweight, as well as infant mortality and morbidity (Hussain et al. 2015). Similarly, poor maternal physical and mental health before, during and after birth has negative effects on child nutrition and food security (Holland and Rammohan 2019). Beyond factors related to maternal health, the literature also highlights the importance of appropriate breastfeeding and complementary feeding practices (Black et al. 2013; Stewart et al. 2013). According to WHO recommendations, infants should be exclusively breastfed until the age of 6 months, when they should receive a varied, nutrient‐rich diet while continuing to be breastfed until the age of 2. This practice promotes better nutritional status for children and protects mothers from early pregnancy (World Health Organization 2003). In addition, other factors have been identified such as domestic violence, age, mother's education and household income (Ackerson and Subramanian 2008; Duflo 2012b; Imai et al. 2014).

Another strand of the literature recognizes that the mother's ability to direct or decide on the allocation of resources within the household is an important factor in children's nutritional status (Doss 2013; Thomas 1997). Indeed, compared to men, women are more likely to devote household resources to subsistence and nutrition, including the well‐being of their children (Dwyer and Bruce 1993). Mothers are the first to be entrusted with the responsibility of caring for children, and in fulfilling this role, the more resources they have access to, the greater the proportion they are able to devote to their care and nutritional needs. This ability of women to allocate more resources to spending on children depends on the negotiation process and decision‐making power they hold within the household (McKenna et al. 2019). Thus, women's decision‐making power within the household appears to be a factor favoring adequate nutrition, both in quantity and quality, for children (McKenna et al. 2019). In the empirical literature, women's decision‐making power is defined as their ability to make decisions freely and independently or jointly, particularly in areas concerning their well‐being and that of their children (Amoak et al. 2025; Anbesu et al. 2023).

Numerous empirical studies have explored the relationship between women's empowerment and children's nutritional status (Abreha and Zereyesus 2021; Cunningham et al. 2015; Malapit et al. 2015). Depending on the dimensions of women's empowerment considered, it sometimes shows a significant association with children's nutritional status, and sometimes a non‐significant association (Kamiya et al. 2018; Shroff et al. 2009; Yaya et al. 2020). In addition, some research has specifically examined the effect of women's decision‐making power, considered a direct measure of empowerment (Kabeer 1999). Thus, studies such as those by Lépine and Strobl (2013), Salman et al. (2023), and Aminou and Monwanou (2022) found a negative and significant association between women's decision‐making autonomy and unfavorable nutritional indicators in children (wasting, stunting and underweight). Other works found no significant effect of women's decision‐making power on children's nutritional status (McKenna et al. 2019; Rajaram et al. 2017). McKenna et al. (2019) show that no dimension of women's decision‐making power is associated with stunting or wasting in children. Similarly, some studies reveal a limited influence of women's decision‐making autonomy on children's nutritional outcomes (Brunson et al. 2009; Dancer and Rammohan 2009). These studies used an approach in which analysis of women's decision‐making power depends entirely on their responses.

Similarly, much of the literature on developing countries uses conventional anthropometric indicators, including underweight (low weight‐for‐age), stunting (low height‐for‐age), and wasting (low weight‐for‐height) as measures of child malnutrition. These conventional indicators of malnutrition are also commonly used in studies conducted in Benin (Addo et al. 2023; Noudamadjo et al. 2023). However, these measures do not provide a complete estimate of the proportion of undernourished children in the population (Nandy and Svedberg 2012; Vollmer et al. 2017). To address this limitation, an alternative measure known as the Composite Index of Anthropometric Failure (CIAF) has been proposed (Svedberg 2000) and used in recent studies to assess the prevalence of malnutrition among children (Endris et al. 2017; Khan and Raza 2016; Vollmer et al. 2017). Studies have also indicated that children with multiple anthropometric deficiencies are at higher risk of early mortality (Khan and Das 2020; McDonald et al. 2013).

Going beyond the existing literature, this study combines a dyadic measure of women's decision‐making power based on cross‐referenced responses from both spouses on household decision‐making with the CIAF to provide new evidence on child malnutrition in Benin. This approach links intra‐household power dynamics including claimed power, recognized power, and effective power resulting from mutual agreement to a global measure of malnutrition, which, to our knowledge, has not yet been explored in this context.

The rest of this research is organized as follows: Section 2 describes the data and variables used in the study, Section 3 presents the study's method, Section 4 reports the results, and Section 5 presents the discussion. Section 6 provides robustness checks, Section 7 discusses the study's limitations and strengths, and Section 8 concludes the study.

## 2 Data and Variables

### 2.1 Data

The data used come from the fifth Benin Demographic and Health Survey (EDSB‐V), conducted from November 6, 2017 to February 28, 2018 among 14,435 households. This is a major national survey of a representative sample of households, covering issues such as the nutritional status of mothers and children, prenatal care, skilled delivery services, postnatal care, child immunization, maternal and child deaths, HIV/AIDS, domestic violence and women's empowerment (INSAE & ICF International 2019). Five modules make up the survey: the women's module, the men's module, the couple's module, the children's module and the household module. Mothers aged between 15 and 49 years completed the children's module resulting in a total sample of 13,589 children. While the children's module is the main module used in this analysis, responses from women and their spouses concerning women's decision‐making role were cross‐referenced from the couple's module and merged with the children's module to obtain the information required for analysis. The unit of analysis is children under 5 years of age born to married or cohabiting couples. The final sample comprises 4,093 children.

### 2.2 Variables

The dependent variable is children's nutritional status. In the literature, children's nutritional status is generally measured using three anthropometric indicators, namely weight‐for‐age Z‐score (WAZ), weight‐for‐height Z‐score (WHZ) and height‐for‐age Z‐score (HAZ) (Wang and Chen 2012; World Health Organization 2006). From these indicators, four groups of childhood malnutrition are obtained: underweight, stunting, wasting and overweight. Stunting, or height too low for age, is a measure of chronic malnutrition, generally caused by poor diet over a long period or by chronic, recurrent illness. Wasting, or weight too low for height, is an indicator of acute malnutrition, often linked to poor diet over a recent period. Underweight combines the effects of chronic and acute malnutrition. Each indicator is obtained by calculating a Z‐score of less than −2 standard deviations (SD) from the median of the reference population. Thus, stunting is a binary variable that takes the value 1 if the HAZ < −2 SD and 0 otherwise (Warssamo et al. 2025). Underweight is a binary variable that takes the value 1 if the WAZ < −2 SD and 0 otherwise (Rahman et al. 2026). Wasting is a binary variable that takes the value 1 if the WHZ < −2 SD and 0 otherwise (Amugsi et al. 2026).

However, these three indicators can't be used to estimate the overall prevalence of child malnutrition in a population. For example, underweight considered as a single relevant indicator of childhood malnutrition does not allow the identification of children cumulating underweight with stunting and or wasting (Nandy and Svedberg 2012). To remedy this limitation, an alternative indicator of malnutrition in young children, namely the CIAF, has been developed to measure the overall burden of childhood malnutrition. The CIAF is composed of several groups or combinations of anthropometric failures (Svedberg 2000; Nandy et al. 2005). According to this CIAF classification, children are divided into seven groups: Group A, no failure; Group B, wasting only; Group C, wasting and underweight; Group D, wasting, stunting and underweight; Group E, stunting and underweight; Group F, stunting only (Svedberg 2000) and Group Y, underweight only (Nandy et al. 2005). This classification is presented in Table 1. The CIAF is a binary variable that takes the value 1 if the child has at least one of the three deficiencies (stunting, underweight or wasting), alone or in combination, and 0 otherwise (Vollmer et al. 2017).

**Table 1:** Groups used to construct the Composite Index of Anthropometric Failure (CIAF) for children.

Group	Description	Wasting	Stunting	Underweight
A	No failure: children have normal height and weight for their age	No	No	No
B	Wasting only: children have abnormal weight for their height	Yes	No	No
C	Wasting and underweight: Children have both an abnormal weight for their height and an abnormal weight for their age.	Yes	No	Yes
D	Wasting, stunting, and underweight: children have both low height and low weight for their age, as well as abnormal weight for their height	Yes	Yes	Yes
E	Stunting and underweight: children have both low height and low weight for their age	No	Yes	Yes
F	Stunting only: children are only too short for their age	No	Yes	No
Y	Underweight only: children have only low weight for their age	No	No	Yes

To construct the indicator of children's nutritional status, we therefore used the conventional measures of malnutrition, namely underweight (low weight‐for‐age), wasting (low weight‐for‐height) and stunting (low height‐for‐age) presented in Appendix Table A1.

The variable of interest (women's decision‐making power within the household) is measured initially on the basis of women's statements regarding their involvement (alone or jointly with their husband) in household decision‐making in two domains: major household purchases and their own healthcare. Secondly, it is assessed by cross‐referencing responses from women and their husbands, broken down as follows: only the woman asserts that she decides (power seized by the woman), only the husband asserts that the woman decides (power granted by the husband), the woman and her husband agree that she decides (agreement on the woman's involvement), and the woman and her husband agree that she does not decide (agreement on the woman's non‐involvement) *(*Annan et al. 2021). This second approach is only for decision‐making regarding major household purchases.

Table 2 presents the various decision‐making scenarios based on the interactions between spouses' responses. These response categories are grouped to indicate whether the woman is involved in decision‐making or not.

**Table 2:** Interaction of responses from both spouses on the woman's involvement in decision‐making.

Person who usually decides on major household purchases		Women's responses	
		The woman decides	The woman does not decide
Husbands's responses	The woman decides	Agreement on the woman's involvement	Power granted by the husband
	The woman does not decide	Power seized by the woman	Agreement on the woman's non‐involvement

The “Agreement on the woman's non‐involvement” variable takes the value 1 when the woman and her husband agree she is not involved in decision‐making, and 0 otherwise. The “Agreement on the woman's involvement” variable takes the value 1 when both the woman and her husband agree she is involved in decision‐making, and 0 otherwise. The “Power seized by the woman” variable takes the value 1 when only the woman reports being involved in decision‐making, and 0 otherwise. The “Power granted by the husband” variable takes the value 1 when only the husband states that his wife is involved in decision‐making, and 0 otherwise.

The control variables are grouped into three categories: socio‐demographic characteristics of the child (age, sex, birth order, multiple birth, breastfeeding), demographic, socio‐economic and cultural characteristics of the mother (age at survey, age at first cohabitation, education level, ethnicity, religion, employment status, place of delivery) and household characteristics, namely household size, household wealth index, husband's education, place of residence (Altare et al. 2016; Poda et al. 2017; Sarma et al. 2017).

### 2.3 Ethical Statement

This research is based exclusively on anonymized secondary data from the Benin Demographic and Health Survey (DHS), for which ethical approval was obtained from the relevant ethics committees. The study therefore adheres to established ethical principles governing research with publicly available, de‐identified data.

## 3 Method

The objective of this analysis is to examine the effect of women's decision‐making power within the household on children's nutritional status. Decision‐making power is measured using different indicators that reflect women's ability to participate in household decisions. Children's nutritional status is measured by the CIAF, an indicator that provides a comprehensive estimate of the number of malnourished children in the population.

Referring to the literature on the determinants of children's nutritional status (Permatasari and Chadirin 2022; Workie and Tesfaw 2021), we specify the following equation:

$$Y_{i}^{*}=\beta X_{i}+\delta D_{i}+\varepsilon_{i}$$

where $Y_{i}$ represents children's nutritional status measured by the CIAF. $X_{i}\;$is a vector of exogenous variables consisting of child, mother and household characteristics. $D_{i}$ is a variable representing women's decision‐making power. $\varepsilon_{i}$ is the error term. βand δ are the coefficients to be estimated. Since the dependent variable is dichotomous, Equation (1) can be estimated using a simple probit model. However, the estimates will be biased if women's decision‐making power is endogenous.

Women's decision‐making may be subject to endogeneity bias, as highlighted in the literature (Haider et al. 2017; Kamiya 2011; Lépine and Strobl 2013). Two main sources of endogeneity are generally identified: unobserved heterogeneity (unmeasured factors that simultaneously influence women's decision‐making power and children's nutritional status) and reverse causality (where children's nutritional status may influence women's decision‐making power). However, in empirical studies using cross‐sectional data, reverse causality is generally recognized as a possibility but is not directly assessed (Bliznashka et al. 2021; McKenna et al. 2019; Yaya et al. 2020). In this study, we consider that endogeneity stems primarily from unobserved factors that also influence children's nutritional status. Our identification strategy therefore involves remedying this source of endogeneity.

Within the framework of this identification strategy, we use a simultaneous equations model to correct this endogeneity. With this type of model, the endogenous variable can be continuous, discrete (Heckman 1977) or dichotomous (Maddala 1983). In this study, the outcome variable (children's nutritional status) is dichotomous, as is the potentially endogenous variable (women's decision‐making power). Estimation using a recursive bivariate probit model therefore appears most appropriate (Kamiya 2011). Indeed, the recursive bivariate probit model can be used in the presence of bias due to unobserved heterogeneity, when unobserved factors are correlated with both the binary endogenous variable and the binary outcome variable (Mboutchouang et al. 2023). The model is formalized as follows:

(1) $$Y_{i}^{*}=\beta X_{i}+\delta D_{i}+\varepsilon_{i}$$

(2) $$D_{i}^{*}=\alpha X_{i}^{\prime }+\gamma Z_{i}+\mu_{i}$$

where $Y_{i}^{*}$ and $D_{i}^{*}$ represent the latent variables of the child's nutritional status (measured by the CIAF) and the decision‐making power of woman i, respectively. $X_{i}^{\prime }$ is a vector of exogenous variables describing mother and household characteristics, including the mother's age, the mother's and father's education levels, religion, ethnicity, place of residence, household size, and wealth index. $Z_{i}$ is the instrumental variable representing the woman's acceptance of domestic violence. $D_{i}$ is the endogenous variable representing women's decision‐making power. β, δ, α and γ are the parameters to be estimated. The relevance of the instrumental variable is analyzed through a first‐stage diagnostic based on the endogenous variable equation.

The error terms $\varepsilon_{i}$ and $\mu_{i}$ follow a bivariate normal distribution with *E*($\varepsilon_{i}$) = *E*($\mu_{i}$) = 0, Var($\varepsilon_{i}$) = Var($\mu_{i}$) = 1, and Cov($\varepsilon_{i}\;,\mu_{i}$) = $\rho_{\varepsilon ,\mu }$, where $\rho_{\varepsilon ,\mu }$ is the correlation coefficient between the error terms of equations [1] and [2]. A test of the nullity of this coefficient ($H_{0}:\;\rho_{\varepsilon ,\mu }=0;\;H_{a}:\;\rho_{\varepsilon ,\mu }\neq 0)$ will determine the existence of an endogeneity bias. If the null hypothesis is accepted ($\rho_{\varepsilon ,\mu }$ statistically insignificant), then women's decision‐making power is exogenous, and a simple probit model is sufficient. On the other hand, if the alternative hypothesis is accepted, then there is indeed an endogeneity bias and the recursive bivariate probit model is required (Waters 1999).

To address the endogeneity of women's decision‐making power, we use women's acceptance of domestic violence as an instrumental variable. Domestic violence is rooted in patriarchal norms and unequal power relations within the household that contribute to maintaining women in a position of subordination (Dobash and Dobash 1979). From this perspective, the acceptance of domestic violence may reflect an internalization of these norms, which reduces women's bargaining power and their autonomy within the household. This relationship is consistent with empirical evidence that indicates an association between attitudes toward wife beating and women's decision‐making autonomy (Kirkwood et al. 2024; Michael and Naidoo 2024).

The exclusion constraint is that women's acceptance of domestic violence influences the CIAF only through their decision‐making power. Although the empirical evidence remains mixed (Baruda et al. 2022; Mekonnen et al. 2021), the absence of a link highlighted by some studies (Baruda et al. 2022; Ickes et al. 2015) supports the idea that women's decision‐making power is the pathway through which their acceptance of domestic violence influences children's anthropometric outcomes.

## 4 Results

### 4.1 Descriptive Statistics

Table 3 shows that, according to the different anthropometric deficiency groups, more than half of the children (64.24%) showed no deficiency at all, and thus belonged to group A. On the other hand, 1.33% of children were wasted (group B), 1.57% were wasted and underweight (group C), and 1.70% were wasted, stunted and underweight (group D). The highest prevalence of malnutrition was observed in group F (stunting only) with 19.16%, followed by group E (stunting and underweight) with 10.69%. However, the prevalence of malnutrition is lowest, defined as underweight only (group Y) at 1.30%.

**Table 3:** Groups of anthropometric failure among Benin children, under 5.

Groups of anthropometric failure	Percentage
A: No failure	64.24
B: Wasting only	1.33
C: Wasting + Underweight	1.57
D: Wasting + Stunting + Underweight	1.70
E: Stunting + Underweight	10.69
F: Stunting only	19.16
Y: Underweight only	1.30
Total	100

Table 4 shows that, according to conventional malnutrition indicators, 15.27% of children are underweight, 31.56% stunted and 4.60% wasted. The CIAF indicates a higher prevalence of anthropometric failure, reaching 35.76%. With regard to the variables of interest, the statistics show that 40.95% of children have mothers who are involved in decisions concerning major household purchases, and 40.64% have mothers involved in decisions concerning healthcare. In addition, analysis of spouses' cross‐tabulated responses on women's ability to make decisions about major household purchases shows that 35.54% of children have mothers with non‐involvement in such decisions, and 23.52% have mothers to whom decision‐making power is mainly granted by the husband. 23.04% have mothers who seize the decision‐making power for themselves. Only 17.91% of children live in households where the spouses agree on the woman's involvement.

**Table 4:** Descriptive statistics.

Variables	Mean	Standard deviation	Min	Max
Indicators of children's nutritional status				
Underweight (WAZ < −2)	0.1527	0.3642	0	1
Stunting (HAZ < −2)	0.3156	0.4648	0	1
Wasting (WHZ < −2)	0.0460	0.2095	0	1
Composite Index of Anthropometric Failure (CIAF)	0.3576	0.4794	0	1
Woman's decision‐making power regarding major household purchases				
Woman's responses only				
Woman does not decide	0.5905	0.4918	0	1
Woman decides	0.4095	0.4918	0	1
Interaction of spouses' responses				
Agreement on the woman's non‐involvement	0.3554	0.4787	0	1
Power seized by the woman	0.2304	0.4211	0	1
Power granted by the husband	0.2352	0.4242	0	1
Agreement on the woman's involvement	0.1791	0.3835	0	1
Woman's decision‐making power regarding her own healthcare				
Woman's responses only				
Woman does not decide	0.5936	0.4912	0	1
Woman decides	0.4064	0.4912	0	1
Child characteristics				
Child's age (in months)	27.92	17.59	0	59
Child's sex				
Male	0.5056	0.5000	0	1
Female	0.4944	0.5000	0	1
Birth order	3.588	2.204	1	14
Multiple birth				
Singleton	0.9567	0.2036	0	1
Multiple	0.0433	0.2036	0	1
Breastfeeding				
Breastfed	0.9625	0.1901	0	1
Not breastfed	0.0375	0.1901	0	1
Mother's characteristics				
Mother's age	29.3259	6.4625	15	49
Age at first cohabitation	18.3107	4.3026	10	44
Education				
None	0.6752	0.4684	0	1
Primary	0.1794	0.3837	0	1
Secondary and above	0.1454	0.3525	0	1
Employment status				
Unpaid	0.3206	0.4668	0	1
Paid	0.6794	0.4668	0	1
Religion				
Muslim	0.3689	0.4826	0	1
Christian	0.4733	0.4993	0	1
Other	0.1578	0.3646	0	1
Ethnicity				
Adja & related	0.1084	0.3110	0	1
Bariba & related	0.1361	0.3429	0	1
Dendi & related	0.0682	0.2520	0	1
Fon & related	0.3181	0.4658	0	1
Yoa, Lokpa & related	0.0349	0.1835	0	1
Betamaribe & related	0.0659	0.2481	0	1
Peulh & related	0.1122	0.3157	0	1
Yoruba & related	0.1009	0.3013	0	1
Other	0.0554	0.2287	0	1
Place of delivery				
Home	0.1458	0.3530	0	1
Hospital	0.8542	0.3530	0	1
Household characteristics				
Household size	7.734	4.522	3	40
Husband's education				
None	0.5067	0.5000	0	1
Primary	0.2398	0.4270	0	1
Secondary and above	0.2535	0.4351	0	1
Household wealth index				
Poor	0.4386	0.4963	0	1
Medium	0.1906	0.3928	0	1
Rich	0.3708	0.4831	0	1
Place of residence				
Urban	0.3995	0.4899	0	1
Rural	0.6005	0.4899	0	1
Instrumental variable				
Woman's acceptance of domestic violence				
No	0.6509	0.4767	0	1
Yes	0.3491	0.4767	0	1

A bivariate analysis of the distribution of women's decision‐making power in relation to the CIAF (see Table A2) shows that, in the sample, the proportion of malnourished children is higher when decision‐making power is granted by the husband (38.97%) and lower when both spouses agree on the woman's involvement in decision‐making (32.92%). The remaining situations fall at intermediate levels, with 36.64% when the spouses agree on the woman's non‐involvement and 33.34% when the woman seizes the decision‐making power for herself.

### 4.2 Econometric Results

#### 4.2.1 Diagnostic Tests

Before reporting the main results, we first assess the validity of our identification strategy. Specifically, we test the endogeneity of the variables representing women's decision‐making power in the CIAF equation. The Wald test shows that the exogeneity hypothesis is rejected only when the woman's decision‐making power is granted by the husband. For all other decision‐making variables, there is no significant evidence of endogeneity bias (see Appendix Table A3). Similarly, the first‐stage diagnostic (reported in Appendix Table A4) shows that a woman's acceptance of domestic violence has a positive and significant influence on her decision‐making power (particularly the power granted by her husband). This confirms the relevance of the instrument. Based on this, we estimate a recursive bivariate probit model in which decision‐making power granted by the husband is taken as the endogenous variable and a simple probit model for the other variables to decision‐making power.

#### 4.2.2 Main Results

Table 5 presents the marginal effects of women's decision‐making power over major household purchases on children's nutritional status. Overall, women's decision‐making power does not significantly reduce the probability of experiencing anthropometric failure among children. In particular, when decision‐making power is seized by women or results from agreement between spouses on the women's involvement, the observed effect is negative but statistically insignificant. However, when decision‐making power is granted to women by their husbands, the probability of anthropometric failure among children increases.

**Table 5:** Effect of women's decision‐making power regarding major household purchases on CIAF.

Variables	CIAF		
	Model 1 (Probit)	Model 2 (Probit)	Model 3 (Biprobit)
Woman's decision‐making power regarding major household purchases			
Woman's responses only (Model 1)			
Woman decides (Reference=woman does not decide)	−0.0135		
	(0.0170)		
Interaction of spouses' responses (Models 2‐3)			
Spousal agreement:			
Agreement on the woman's non‐involvement (Reference)			
Agreement on the woman's involvement		−0.0119	−0.0015
		(0.0238)	(0.0028)
Spousal disagreement			
Power seized by the woman		−0.0013	−0.0005
		(0.0216)	(0.0025)
Power granted by the husband		0.0204	0.0594***
		(0.0211)	(0.0033)
Child's age (in months)	0.0022***	0.0022***	0.0003***
	(0.0005)	(0.0005)	(0.0001)
Child's sex (ref= Male)			
Female	−0.0736***	−0.0738***	−0.0081***
	(0.0156)	(0.0156)	(0.0029)
Birth order	0.0190***	0.0189***	0.0022**
	(0.0069)	(0.0069)	(0.0010)
Multiple birth (ref = Singleton)	0.2007***	0.2009***	0.0253***
	(0.0417)	(0.0416)	(0.0097)
Mother's age	−0.0089***	−0.0089***	−0.0012***
	(0.0025)	(0.0025)	(0.0004)
Age at first cohabitation	0.0021	0.0021	0.00005
	(0.0024)	(0.0024)	(0.0004)
Household size	0.0027	0.0028	−0.0001
	(0.0019)	(0.0019)	(0.0003)
Mother's education (ref = None)			
Primary	0.0030	0.0030	0.0011
	(0.0233)	(0.0233)	(0.0043)
Secondary and above	−0.0265	−0.0272	0.0028
	(0.0276)	(0.0276)	(0.0049)
Paid employment (ref = unpaid)	−0.0153	−0.0148	−0.0175***
	(0.0186)	(0.0186)	(0.0044)
Religion (ref = Other)			
Muslim	−0.0349	−0.0352	−0.0018
	(0.0309)	(0.0309)	(0.0053)
Christian	−0.0205	−0.0210	0.0023
	(0.0244)	(0.0244)	(0.0043)
Ethnicity (ref =Bariba & related)			
Adja & related	0.0621*	0.0632*	0.0015
	(0.0369)	(0.0368)	(0.0074)
Dendi & related	0.0233	0.0256	−0.0175***
	(0.0375)	(0.0376)	(0.0059)
Fon & related	0.0824***	0.0857***	−0.0228***
	(0.0316)	(0.0317)	(0.0061)
Yoa, Lokpa & related	0.0488	0.0517	−0.0153**
	(0.0463)	(0.0464)	(0.0072)
Betamaribe & related	0.0097	0.0128	−0.0195***
	(0.0373)	(0.0373)	(0.0066)
Peulh & related	0.1131***	0.1125***	0.0240**
	(0.0329)	(0.0328)	(0.0110)
Yoruba & related	0.0271	0.0292	−0.0125**
	(0.0345)	(0.0345)	(0.0058)
Others	0.0712*	0.0742*	−0.0127*
	(0.0381)	(0.0382)	(0.0068)
Hospital delivery (ref = home delivery)	−0.0733***	−0.0739***	−0.0082**
	(0.0250)	(0.0250)	(0.0037)
Non‐breastfed child (ref = breastfed child)	0.0674*	0.0648	0.0065
	(0.0401)	(0.0401)	(0.0051)
Husband's education (ref = None)			
Primary	−0.0195	−0.0202	0.0015
	(0.0210)	(0.0211)	(0.0040)
Secondary and above	−0.0230	−0.0237	0.0039
	(0.0239)	(0.0240)	(0.0044)
Household wealth index (ref = Poor)			
Medium	−0.0367	−0.0366	−0.0061
	(0.0228)	(0.0228)	(0.0044)
Rich	−0.1068***	−0.1064***	−0.0177***
	(0.0230)	(0.0230)	(0.0049)
Rural (ref = urban)	−0.0064	−0.0068	0.0022
	(0.0177)	(0.0177)	(0.0034)
Number of observations	4,093	4,093	4,093
Prob > chi2	0.0000	0.0000	0.0000
Log pseudolikelihood	−2582.4531	−2581.802	−4663.5351
Pseudo *R*2	0.0441	0.0443	

In other words, children living in households where women's decision‐making power over major household purchases is mainly granted by their husbands have a 5.94 percentage points higher probability of experiencing anthropometric failure than those living in households where women have no decision‐making power.

Table 6 presents the marginal effects of women's decision‐making power regarding their healthcare on the CIAF. The results show that this variable has no significant effect on the probability of anthropometric failure among children.

**Table 6:** Effect of women's decision‐making power regarding their healthcare on CIAF.

Variables	CIAF
	Probit model
Woman's decision‐making power regarding her healthcare	
Woman's responses only	
Woman decides (Reference = woman does not decide)	−0.0201
	(0.0169)
Child's age (in months)	0.0022***
	(0.0005)
Child's sex (ref= Male)	
Female	−0.0734***
	(0.0156)
Birth order	0.0188***
	(0.0069)
Multiple birth (ref = Singleton)	0.2016***
	(0.0416)
Mother's age	−0.0088***
	(0.0025)
Age at first cohabitation	0.0020
	(0.0024)
Household size	0.0027
	(0.0019)
Mother's education (ref = None)	
Primary	0.0023
	0.0233)
Secondary and above	−0.0271
	(0.0275)
Paid employment (ref = unpaid)	−0.0136
	(0.0186)
Religion (ref = Other)	
Muslim	−0.0353
	(0.0309)
Christian	−0.0201
	(0.0244)
Ethnicity (ref =Bariba & related)	
Adja & related	0.0632*
	(0.0369)
Dendi & related	0.0230
	(0.0376)
Fon & related	0.0817***
	(0.0316)
Yoa, Lokpa & related	0.0499
	(0.0464)
Betamaribe & related	0.0103
	(0.0373)
Peulh & related	0.1130***
	(0.0329)
Yoruba & related	0.0266
	(0.0345)
Others	0.0705*
	(0.0381)
Hospital delivery (ref = home delivery)	−0.0728***
	(0.0250
Non‐breastfed child (ref = breastfed child)	0.0668*
	(0.0401)
Husband's education (ref = None)	
Primary	−0.0195
	(0.0210)
Secondary and above	−0.0228
	(0.0239)
Household wealth index (ref = Poor)	
Medium	−0.0369
	(0.0228)
Rich	−0.1064***
	(0.0230)
Rural (ref = urban)	−0.0060
	(0.0177)
Number of observations	4,093
Prob > chi2	0.0000
Log pseudolikelihood	−2582.0032
Pseudo *R* ^2^	0.0443

*Note:* * and *** indicate statistical significance at the 10% and 1% levels, respectively. Values in parentheses represent standard deviations. Source: Author, based on 2017–2018 Benin DHS data.

## 5 Discussion

Our study uses cross‐referenced responses from wives and husbands on women's decision‐making power within the household, combined with the CIAF to provide new evidence on child malnutrition in Benin. In our sample, nearly half of the couples (46.56%) report disagreement regarding decision‐making. We find that children whose mothers are granted decision‐making power by their husbands face a higher probability of experiencing anthropometric failure. This result is consistent with the descriptive statistics, which also show that the prevalence of malnourished children is higher in households where decision‐making power is attributed to the woman by her husband. One possible explanation is that women to whom decision‐making power is granted may not have real control over household resources. Receiving power, or what some authors refer to as empowerment granted by another person or group, could mask an attempt to retain control over resources (Taliaferro 1991). As several authors point out, women's effective control over resources improves children's health outcomes (Pratley 2016). Moreover, classical theories of power in the social and political sciences emphasize individuals' recognition and assertion of their own rights and influence. O'Hara and Clement (2018) indicate that individuals who recognize and claim their own capacity are more autonomous than those to whom this capacity is attributed by others. However, these mechanisms, notably the transfer of responsibility without resources, symbolic empowerment and women's lack of effective control over resources, which have been put forward in the literature as plausible explanations for the observed association, were not directly assessed in this study.

However, women's decision‐making power regarding their healthcare has no significant effect on children's anthropometric failure. This result is consistent with the findings of Asif (2019) in Pakistan, who also show that mothers' decision‐making, whether independent or joint, is not significantly associated with child undernutrition as measured by the CIAF. Similarly, using a composite index, Khan and Raza (2019) find that women's empowerment does not significantly influence children's nutritional status in Bangladesh, as measured by the CIAF.

Other individual and household characteristics also influence children's nutritional status. In particular, a child's age has a positive and significant effect on the probability of anthropometric failure. As age increases, the likelihood of experiencing anthropometric failure also increases. This is because, as children grow, they require complementary feeding in addition to breastfeeding to support their growth. The WHO recommends exclusive breastfeeding for children under 6 months of age and complementary feeding alongside breastfeeding up to the age of 2 years and beyond. Until the age of 5, children undergo a process of physical and mental growth that requires a healthy and balanced diet (Islam and Biswas 2020). This finding is consistent with several studies conducted in different countries (Islam and Biswas 2020; Khamis et al. 2020). In Tanzania, Khamis et al. (2020) indicate that age strongly influences malnutrition among children. They show that children aged 0 to 6 months have a significantly lower anthropometric prevalence than children aged 6 months. In contrast, Workie and Tesfaw (2021) find that, in Ethiopia, a child's age is not significantly associated with the probability of experiencing anthropometric failure.

Birth order is positively and significantly associated with the probability of experiencing anthropometric failure. Indeed, as birth order increases, so does the number of children in the household, which reduces the resources available for child care and nutrition. Breastfeeding also has a significant influence on anthropometric failure among children. Children who were not breastfed have a higher probability of experiencing anthropometric failure compared to children who were breastfed. Breast milk contains the nutrients necessary to maintain adequate nutritional status during early childhood.

Delivery in a health facility has a significant negative effect on the probability of experiencing anthropometric failure. Children born in health facilities are less likely to suffer from anthropometric failure than those born at home. This is because mothers who give birth in health facilities receive appropriate obstetric care and the information necessary for the health and well‐being of the newborn. Similar findings were reported in a study conducted in Myanmar, Southeast Asia (Kang and Kim 2019). Likewise, household wealth negatively affects the probability of experiencing anthropometric failure among children. Indeed, children from wealthy households are less likely to suffer from anthropometric failure. This is because better household living conditions promote adequate access to food and healthcare services. This result is consistent with the findings of Endris et al. (2017) in Ethiopia, Islam and Biswas (2020) in Bangladesh, and Vollmer et al. (2017), who report that household wealth affects the probability of anthropometric failure.

## 6 Robustness Checks

To assess the robustness of our results, we use an alternative indicator of malnutrition, namely stunting. In our sample, descriptive statistics for standard malnutrition indicators show that 15.27% of children under 5 are underweight, 31.56% are stunted, and 4.60% are wasted. The prevalence of stunting is therefore higher among children under 5. This indicator of chronic malnutrition reflects inadequate nutrition over a long period. Accordingly, we use this indicator to analyze its relationship with women's decision‐making power. The results of the robustness analysis are presented in Appendix Tables A5 and A6.

According to women's self‐reports, the results indicate that women's decision‐making power has no significant effect on the probability of stunting among children. However, when the spouses' cross‐referenced responses are taken into account, it becomes clear that a woman's decision‐making power has a significant effect only when it is attributed to her by her spouse. In addition, a woman's participation in decision‐making regarding her own healthcare does not significantly influence the probability of the child being stunted. These new insights revealed by the robustness checks lead to the same conclusions as those formulated previously, therefore confirming the robustness of our results.

## 7 Limitations and Strengths

The study has some limitations. First, the cross‐sectional nature of the data used does not allow for the establishment of causal relationships between women's decision‐making power and children's nutritional status. Thus, the results should be interpreted solely as associations. Second, questions regarding certain aspects of decision‐making within the household were directed exclusively at women, which prevents the construction of dyadic indicators of decision‐making power across all the domains considered. Nevertheless, this study makes a significant contribution to the literature by linking intra‐household power dynamics using a dyadic approach that includes claimed power, recognized power, and power derived from mutual agreement, to an overall measure of child malnutrition. This provides new empirical evidence regarding the influence of intra‐household decision‐making power on children's nutritional status.

## 8 Conclusion

The main objective of this article was to analyze the effect of women's decision‐making power within the household on the nutritional status of children under 5 in Benin. The data come from the 2017–2018 Benin Demographic and Health Survey. The sample included 4093 children under 5 years of age. Of these, 35.76% were classified as malnourished according to the CIAF. We used this indicator because of its ability to estimate an overall measure of child malnutrition in the population. Decision‐making power was measured using two approaches. The first considers only women's responses regarding their ability to participate in household decision‐making. The second approach considers cross‐referenced responses from spouses on women's decision‐making power. The econometric analysis is performed using simple probit and recursive bivariate probit models to account for the potential endogeneity bias of decision‐making power.

The results of descriptive statistics show that the prevalence of CIAF is 35.76%, which is higher than the prevalence reported by conventional anthropometric indicators of underweight (15.27%), stunting (31.56%), and wasting (4.60%). The estimation results from the simple probit and recursive bivariate probit models reveal that, regarding women's responses, their participation in decision‐making on major household purchases and their healthcare has no significant effect on the probability of experiencing anthropometric failure among children. However, taking into account the interaction of spouses' responses shows that decision‐making power seized by women and resulting from agreement between spouses on the women's involvement have negative but statistically insignificant effects on this probability. Furthermore, the probability of children experiencing anthropometric failure increases by 5.94 percentage points in households where women's decision‐making power is granted to them by their husbands. In addition, female children, children of older mothers, children from wealthier households, and children born in health facilities are less likely to experience anthropometric failure.

Overall, women's decision‐making power does not significantly reduce the probability of experiencing anthropometric failure among children, but it can increase this probability when the husband grants decision‐making power to his wife. This type of power, which reflects a disagreement between spouses regarding decision‐making within the household, undercuts, rather than improve women's ability to influence their children's nutritional status. The authority granted by the husband may involve a transfer of responsibility in a context of economic vulnerability. Thus, a woman may be recognized as a decision‐maker but still lack sufficient resources, namely the financial or material means, to ensure adequate food for her children or to improve their nutritional status. Policies aimed at strengthening women's decision‐making power should be accompanied by mechanisms to improve access to and control over economic resources, so that this power may lead to better child nutritional status.

## Author Contributions

The author conceptualized the study, developed the methodology, conducted the data analysis, interpreted the findings, and wrote and revised the manuscript. The author read and approved the final version.

## Funding

The author has nothing to report.

## Conflicts of Interest

The author declares no conflicts of interest.

## Data Availability

The data that support the findings of this study are available from the corresponding author upon reasonable request.

## 1

**Table A1:** Child malnutrition indicators.

Variables	Codes
Underweight	1 includes children with weight‐for‐age Z‐scores (WAZ) < −2 standard deviations from the WHO child growth standard median, 0 otherwise
Stunting	1 includes children with height‐for‐age Z‐scores (HAZ) < −2 standard deviations from the WHO child growth standard median, 0 otherwise
Wasting	1 includes children with weight‐for‐height Z‐scores (WHZ) < −2 standard deviations from the WHO child growth standard median, 0 otherwise
Composite Index of Anthropometric Failure (CIAF)	1 if children present one or more anthropometric failures (wasting only, wasting + underweight, wasting + stunting + underweight, stunting + underweight, stunting only, underweight only), 0 if children have no anthropometric failure

**Table A2:** Descriptive statistics (bivariate).

Variables	Composite index of anthropometric failure (CIAF)		
	Non‐malnourished child	Malnourished child	Pearson chi‐square statistic (*χ* ^2^)
Woman's decision‐making power regarding major household purchases			
Woman's responses only			6.5850***
Woman does not decide	62.43	37.57	
Woman decides	66.85	33.15	
Interaction of responses from both spouses			
Agreement on the woman's non‐involvement	63.36	36.64	1.0525
Power seized by the woman	66.66	33.34	2.5373
Power granted by the husband	61.03	38.97	3.3385*
Agreement on the woman's involvement	67.08	32.92	2.3774
Woman's decision‐making power regarding her own healthcare			
Woman's response only			
Woman does not decide	62.31	37.69	6.2793**
Woman decides	67.05	32.95	

**Table A3:** Significance of correlations between the error terms of the CIAF and women's decision‐making power equations.

Variables	CIAF	
	Rho(ρ)	Wald test (*p‐*value)
Woman's decision‐making power regarding major household purchases		
Woman's responses only		
Woman decides	−0.0247	0.9077
Interaction of spouses’ responses		
Power seized by the woman	0.0180	0.9543
Power granted by the husband	−0.7237	0.0067***
Agreement on woman's involvement	−0.0005	0.9987
Woman's decision‐making power regarding her healthcare		
Woman's responses only	Rho(ρ)	Wald test (*p‐*value)
Woman decides	0.0152	0.9401

**Table A4:** First‐stage diagnostic results.

Variables	Power granted by the husband
Woman's acceptance of domestic violence (ref=No)	0.2303***
	(0.0516)
Mother's age	−0.0021
	(0.0041)
Age at first cohabitation	−0.0049
	(0.0062)
Household size	−0.0101*
	(0.0061)
Mother's education (ref = None)	
Primary	0.0036
	(0.0704)
Secondary and above	0.1214
	(0.0803)
Paid employment (ref = unpaid)	−0.3410***
	(0.0520)
Religion (ref = Other)	
Muslim	0.0296
	(0.0895)
Christian	0.0837
	(0.0724)
Ethnicity (ref =Bariba & related)	
Adja & related	−0.2126**
	(0.1045)
Dendi & related	−0.5000***
	(0.1112)
Fon & related	−0.8022***
	(0.0912)
Yoa. Lokpa & related	−0.5331***
	(0.1390)
Betamaribe & related	−0.5786***
	(0.1085)
Peulh & related	0.1062
	(0.0890)
Yoruba & related	−0.4023***
	(0.1003)
Others	−0.5202***
	(0.1175)
Husband's education (ref = None)	
Primary	0.1088*
	(0.0658)
Secondary and above	0.1503**
	(0.0701)
Household wealth index (ref = Poor)	
Medium	−0.0402
	(0.0653)
Rich	−0.1482**
	(0.0700)
Rural (ref = urban)	0.0778
	(0.0550)
Number of observations	4,093
Prob > chi2	0.0000
Log pseudolikelihood	−4663.5351

**Table A5:** Effect of women's decision‐making power regarding major household purchases on stunting.

Variables			
Woman's decision‐making regarding major household purchases	**Model 1 (Probit)**	**Model 2 (Probit)**	**Model 2 (Biprobit)**
Woman's responses only (Model 1)	−0.0093		
Woman decides (Reference=woman does not decide)	(0.0164)		
Interaction of spouses’ responses (Models 2–3)			
Spousal agreement:			
Agreement on the woman's non‐involvement (Reference)			
Agreement on the woman's involvement		−0.0097	−0.0013
		(0.0229)	(0.0028)
Spousal disagreement			
Power seized by the woman		0.0060	0.0004
		(0.0210)	(0.0026)
Power granted by the husband		0.0229	0.0544***
		(0.0204)	(0.0043)
Child's age (in months)	0.0029***	0.0029***	0.0003***
	(0.0005)	(0.0005)	(0.0001)
Child's sex (ref= Male)			
Female	−0.0667***	−0.0670***	−0.0077***
	(0.0150)	(0.0150)	(0.0029)
Birth order	0.0141**	0.0140**	0.0017*
	(0.0066)	(0.0066)	(0.0009)
Multiple birth (ref = Singleton)	0.2059***	0.2061***	0.0274***
	(0.0415)	(0.0415)	(0.0102)
Mother's age	−0.0068***	−0.0068***	−0.0010**
	(0.0024)	(0.0024)	(0.0004)
Age at first cohabitation	−0.0002	−0.0001	−0.0002
	(0.0023)	(0.0023)	(0.0004)
Household size	0.0036*	0.0037**	0.00003
	(0.0018)	(0.0018)	(0.0003)
Mother's education (ref = None)			
Primary	0.0146	0.0148	0.0020
	(0.0226)	(0.0226)	(0.0041)
Secondary and above	−0.0160	−0.0169	0.0038
	(0.0270)	(0.0270)	(0.0049)
Paid employment (ref = unpaid)	−0.0299*	−0.0294	−0.0181***
	(0.0180)	(0.0180)	(0.0048)
Religion (ref = Other)			
Muslim	−0.0467	−0.0472	−0.0037
	(0.0300)	(0.0300)	(0.0054)
Christian	−0.0339	−0.0345	0.0002
	(0.0236)	(0.0236)	(0.0043)
Ethnicity (ref =Bariba & related)			
Adja & related	0.0575*	0.0586*	0.0027
	(0.0350)	(0.0349)	(0.0071)
Dendi & related	−0.0060	−0.0038	−0.0171***
	(0.0349)	(0.0349)	(0.0060)
Fon & related	0.0959***	0.0995***	−0.0190***
	(0.0303)	(0.0303)	(0.0059)
Yoa, Lokpa & related	0.0522	0.0553	−0.0130*
	(0.0438)	(0.0439)	(0.0068)
Betamaribe & related	0.0190	0.0224	−0.0164**
	(0.0352)	(0.0352)	(0.0065)
Peulh & related	0.1003***	0.0996***	0.0225**
	(0.0311)	(0.0310)	(0.0104)
Yoruba & related	0.0395	0.0415	−0.0094*
	(0.0334)	(0.0333)	(0.0055)
Others	0.0656*	0.0689*	−0.0103
	(0.0366)	(0.0367)	(0.0067)
Hospital delivery (ref = home delivery)	−0.0713***	−0.0720***	−0.0084**
	(0.0242)	(0.0242)	(0.0037)
Non‐breastfed child (ref = breastfed child)	0.0638	0.0608	0.0065
	(0.0390)	(0.0389)	(0.0052)
Husband's education (ref = None)			
Primary	−0.0108	−0.0115	0.0024
	(0.0203)	(0.0203)	(0.0040)
Secondary and above	−0.0357	−0.0364	0.0017
	(0.0230)	(0.0231)	(0.0042)
Household wealth index (ref = Poor)			
Medium	−0.0262	−0.0261	−0.0052
	(0.0223)	(0.0223)	(0.0042)
Rich	−0.1211***	−0.1207***	−0.0190***
	(0.0221)	(0.0221)	(0.0052)
Rural (ref = urban)	−0.0056	−0.0061	0.0023
	(0.0171)	(0.0171)	(0.0033)
Number of observations	4,093	4,093	4,093
Prob > chi2	0.0000	0.0000	0.0000
Log pseudolikelihood	−2434.3861	−2433.3973	−4516.4592
*R* ^2^	0.0577	0.0581	

**Table A6:** Effect of women's decision‐making power regarding their own healthcare on stunting.

Variables	Stunting
Woman's decision‐making power regarding her own healthcare	**Probit model**
Woman's responses only	
Woman decides (Reference = woman does not decide)	−0.0181
	(0.0163)
Child's age (in months)	0.0029***
	(0.0005)
Child's sex (ref= Male)	
Female	−0.0665***
	(0.0150)
Birth order	0.0139**
	(0.0066)
Multiple birth (ref = Singleton)	0.2069***
	(0.0415)
Mother's age	−0.0066***
	(0.0024)
Age at first cohabitation	−0.0002
	(0.0023)
Household size	0.0035*
	(0.0018)
Mother's education (ref = None)	
Primary	0.0141
	(0.0226)
Secondary and above	−0.0166
	(0.0269)
Paid employment (ref = unpaid)	−0.0278
	(0.0180)
Religion (ref = Other)	
Muslim	−0.0471
	(0.0300)
Christian	−0.0333
	(0.0236)
Ethnicity (ref =Bariba & related)	
Adja & related	0.0589*
	(0.0351)
Dendi & related	−0.0062
	(0.0349)
Fon & related	0.0958***
	(0.0303)
Yoa, Lokpa & related	0.0535
	(0.0438)
Betamaribe & related	0.0199
	(0.0353)
Peulh & related	0.1002***
	(0.0311)
Yoruba & related	0.0390
	(0.0334)
Others	0.0651*
	(0.0365)
Hospital delivery (ref = home delivery)	−0.0706***
	(0.0242)
Non‐breastfed child (ref = breastfed child)	0.0630
	(0.0389)
Husband's education (ref = None)	
Primary	−0.0107
	(0.0203)
Secondary and above	−0.0355
	(0.0230)
Household wealth index (ref = Poor)	
Medium	−0.0263
	(0.0223)
Rich	−0.1207***
	(0.0221)
Rural (ref = urban)	−0.0054
	(0.0170)
Number of observations	4,093
Prob > chi2	0.0000
Log pseudolikelihood	−2433.8589
Pseudo *R* ^2^	0.0579
